# Global landscape of cranial prosthesis policies for medical hair loss: a narrative review

**DOI:** 10.3389/fpubh.2026.1870740

**Published:** 2026-07-15

**Authors:** Angela Anaeme, Joey Lu, Caroline Mann

**Affiliations:** 1 Washington University in St. Louis School of Medicine, Saint Louis, MO, United States; 2 Division of Dermatology, John T. Milliken Department of Medicine, Washington University in St. Louis, Saint Louis, MO, United States

**Keywords:** alopecia, cranial prosthesis, durable medical equipment, hair loss, public health, public policy

## Abstract

**Background:**

Cranial prostheses (CPs) provide indispensable cosmetic and emotional support for individuals with medical hair loss. However, access remains inconsistent due to variable reimbursement policies and unclear classification as medical vs. cosmetic devices.

**Objective:**

To characterize global CP coverage frameworks and evaluate how each predominant healthcare system model structures CP access, reimbursement, and eligibility.

**Methods:**

We conducted a narrative review of CP policies across representative countries within the four major healthcare system models: Beveridge, Bismarck, National Health Insurance, and out-of-pocket systems. We synthesized data from peer-reviewed literature, government and insurance policy documents, and nonprofit resources to identify common themes in coverage and access.

**Results:**

CP coverage varies widely across healthcare systems. Beveridge systems provide formal inclusion but demonstrate regional and administrative variability. Bismarck systems range from standardized, diagnosis-based reimbursement to insurer-dependent models with variable out-of-pocket costs. National Health Insurance systems often lack explicit CP coverage, relying on supplemental insurance and charitable support. The United States exhibits a highly fragmented coverage model, while out-of-pocket systems largely lack formal reimbursement pathways. Across all models, oncology-related hair loss is more consistently covered than non-oncologic etiologies.

**Limitations:**

Heterogeneity in available policy data and reliance on publicly available sources may have limited the scope and completeness of our search.

**Conclusion:**

CP coverage is globally inconsistent and frequently insufficient, highlighting the need for standardized recognition of CPs as medically necessary devices to improve equitable access.

## 1 Introduction

Medical hair loss affects millions of individuals worldwide. In fact, alopecia areata (AA) alone is estimated to affect approximately 2% of the global population, while chemotherapy-induced alopecia occurs in an estimated 65–80% of patients receiving cytotoxic chemotherapy (1, 2). Cranial prostheses (CPs), commonly referred to as wigs, are a vital source of support for individuals with hair loss. CPs provide patients with not only direct cosmetic restoration, but also undeniable psychosocial support, with studies consistently showing psychological, emotional, and social benefits of CPs in patients with hair loss (3–6). Additionally, in some cases of hair loss where pharmacologic therapies are costly, ineffective, or contraindicated, CPs may be the most immediate and accessible intervention available (7–9). As such, CPs serve not just as accessories, but as medically indicated devices that mitigate the documented psychosocial burden of hair loss. In health policy and insurance frameworks, a service is generally considered medically necessary when it is required to prevent, diagnose, or treat a condition and improve or maintain health and psychosocial functioning (10, 11). Given the well-established associations between medical hair loss and diminished quality of life, anxiety, depression, and social isolation, CPs may reasonably be viewed as medically necessary interventions that address clinically significant psychosocial sequelae, rather than purely cosmetic concerns (3, 12–14).

Despite their importance, CPs are challenging to access for many patients. Indeed, exorbitant out-of-pocket costs can be restrictive, and reimbursement pathways often only offer partial assistance (12, 15). Even in countries where CP coverage is formally codified, patients often must navigate complex compensation processes, eligibility criteria, and reimbursement caps. In the realm of public policy, CPs occupy an ambiguous position at the intersection of cosmetic and medical devices. This lack of consistency results in variable recognition of CPs in insurance benefit structures.

Part of this inconsistency stems from broader structural differences in how countries finance their healthcare systems and reimburse medical care. CP coverage pathways are shaped by underlying health insurance models, which vary significantly in organization, funding mechanisms, and definitions of medical necessity. Globally, there are four major recognized healthcare system archetypes: Beveridge, Bismarck, National Health Insurance, and out-of-pocket systems (16, 17). Of note, relatively few countries adhere strictly to just one model. Rather, most countries operate within hybrid or mixed-model frameworks (17). Within each of these healthcare systems, there is variability in whether CPs are recognized as reimbursable medical devices, conditionally covered accessories, or entirely excluded from benefit packages.

Given the psychosocial burden of hair loss and the variability in policy surrounding CP reimbursement, a comparative evaluation of international coverage frameworks by healthcare system is warranted. Additionally, improved clinician awareness of coverage pathways may improve counseling and advocacy efforts for patients navigating complex reimbursement processes. In this narrative review, we examine the global landscape of CP coverage across representative countries within the four major healthcare system models. By analyzing how different insurance schema shape access, reimbursement, and eligibility criteria, we aim to clarify existing pathways to CP coverage, and identify policy considerations that may inform future efforts to expand equitable access for individuals with hair loss.

## 2 Methods

We conducted a narrative review of CP coverage across the four major healthcare system models: Beveridge, Bismarck, National Health Insurance, and out-of-pocket systems. We also explore CP coverage in mixed-model systems and the United States in separate discussions, given the unique complexity and heterogeneity of these systems. Although many modern healthcare systems frequently incorporate features of multiple financing models and may not fit neatly into a single category, countries were grouped according to their predominant healthcare financing structure to facilitate comparison and identify broader policy trends. For each healthcare model, three to four representative countries were identified and thoroughly investigated, prioritizing those with publicly documented CP policies or recent legislative developments and initiatives.

A targeted literature search was conducted in PubMed in November 2025. No lower date limit was applied, and all eligible publications indexed in PubMed through the date of the search were considered. Search terms included combinations of “cranial prosthesis,” “wig,” “medical wig,” “hair prosthesis,” “alopecia,” “hair loss,” “coverage,” “reimbursement,” “insurance health policy,” and “healthcare access,” both independently and in combination with individual country names. Searches were performed both broadly and on a country-specific basis to identify peer-reviewed literature describing CP coverage, reimbursement policies, access barriers, psychosocial outcomes, and policy effects. Reference lists of relevant publications were also reviewed to identify potential additional sources.

Representative countries were selected within each healthcare system model based on the availability of publicly accessible information about CP reimbursement policies, geographic diversity, and the presence of notable legislative initiatives or reimbursement frameworks. For each country, supplementary searches were conducted using Google Scholar, general web searches, government health agency websites, national health service resources, statutory insurance documents, legislative records, and publicly available policy materials. These resources were reviewed to identify current CP coverage criteria, reimbursement amounts, eligibility requirements, and coverage limitations. When available, policy information was cross-referenced across multiple sources to improve accuracy and completeness.

To supplement policy information, we also reviewed materials from nonprofit organizations, patient advocacy groups, and community-based organizations involved in supporting individuals with medical hair loss. Sources were included if they contained information related to CP access, reimbursement, eligibility criteria, patient support resources, or policy initiatives. Eligible sources included peer-reviewed publications, government and health agency websites, insurance documents, legislative materials, policy statements, and online publications from reputable nonprofit or advocacy organizations. Sources unrelated to medical hair loss, non-human studies, duplicate records, and materials lacking sufficient policy or clinical relevance were excluded. Two reviewers independently screened sources for relevance and extracted pertinent information. Disagreements regarding source inclusion or data interpretation were resolved through discussion and consensus. All included sources were synthesized qualitatively to identify recurring themes, differences in coverage, and broader policy trends across each healthcare system model.

## 3 Results

### 3.1 Beveridge

The Beveridge healthcare model is characterized by government-funded healthcare financed through general taxation, and delivered primarily through public systems (16). Within this framework, the government determines coverage policies and reimbursement structures with the goal of ensuring universal access and minimizing point of care costs. Countries operating under this model include the United Kingson, Spain, Italy, and Norway (Table 1), though most operate within hybrid adaptations of this model (18–20).

**Table 1:** Cranial prosthesis coverage in countries with Beveridge healthcare systems.

Country	CP policy	Eligibility	Reimbursement	Variation	Key limitations
United Kingdom^a^	Included in NHS	Diagnosis-based; exemptions for children, inpatients, low-income patients	England: predetermined NHS charges (unless exempt) Scotland and Wales: free via hospital supply Renewal period length not defined	High; significant variation depending on region and trusts	Administrative complexity and variability
Spain^b^	CPs not mentioned in national health system list of covered prostheses	No uniform national access pathway; dependent on diagnosis and region	No uniform reimbursement pathway identified, but reimbursed variably by autonomous communities; average €150–€250 per year	High; it is largely patient dependent due to lack of codified guidelines	Ambiguity of national policies; inconsistent access
Italy^c^	Regional regulation exists	Documentation of medical necessity required; oncology-related	No uniform reimbursement pathway, but regional programs exist (€200- €300 yearly)	High; region-specific	No unified national standard
Norway^d^	CPs are included and covered by NAV	Disease or treatment-related hair loss covered with medical documentation	NAV explicitly defines coverage as 6,265 NOK per calendar year	Low; national framework exists	More expensive CPs may not be covered by NAV assistance

^a^National Health Services wig and fabric support guidelines (21); ^b^Spanish Ministry of Health prosthetics services framework (18); ^c^Italian regional health policy (Lombardy) on assistive devices (19); ^d^Norwegian NAV wig policy (20).

Among the Beveridge systems, the United Kingdom provides the most clearly documented framework for CP provision through the National Health Service (NHS). CPs are formally included as “wigs and fabric supports” by the NHS (21, 22). However, access is conditional rather than universal. Patients are typically required to pay standardized charges unless they meet exemption criteria for financial assistance, which include pediatric age ranges (e.g. under 16 years of age, or under 19 years of age in full-time education), inpatient care, or eligibility for income-based assistance programs through the NHS Low Income Scheme or specified benefits (21). Notably, even within this unified national system, access to CPs varies substantially by region. In Scotland and Wales, for instance, CPs are commonly provided free of charge through hospital services, whereas in England, patients are more likely to incur prescription-related costs unless exempt (23). Additional administrative processes, such as reimbursement through the NHS HC5W scheme for individuals who initially pay out-of-pocket but later establish eligibility for assistance, further complicate access pathways (22).

Beyond national policy, local NHS trusts introduce another layer of variability by defining eligibility criteria, replacement frequency, and allowable prosthesis types. Healthcare trusts are regional organizations responsible for the operation of local health services, allowing care to be managed closer to the communities they serve. However, they may introduce an additional layer of complexity to obtaining CP coverage. For example, some trusts limit provision to synthetic CPs and restrict access to one or two products annually depending on the diagnosis, with human hair CPs typically excluded except in rare clinical circumstances. The Royal Devon University Trust in the United Kingdom is one such case, as it specifies that patients undergoing chemotherapy or radiotherapy are entitled to one synthetic CP annually (24).

Another stipulation of CP access in the Beveridge system is diagnosis-specific eligibility, with policies primarily structured around oncology-related hair loss, while guidance for dermatologic or autoimmune forms of alopecia remains limited. NHS CP policies largely cater toward patients with oncologic or hematologic conditions, which may contribute to disparities in access for patients with select forms of hair loss (25). In fact, in a recent 2025 study on NHS CP provision, approximately 50% of clinicians reported difficulty prescribing CPs through NHS pathways, and 11% described the process as impossible (26). The challenge of regional variation held true in this study, with provision being reportedly more straightforward in Scotland and the Southwest of England, yet nearly half of surveyed clinicians in Northern Ireland and London reported that obtaining a prescription CP was “impossible or nearly impossible” (26). Thus, diagnostic prioritization and regional variation both contribute to disparities in access for patients with non-oncologic hair loss conditions.

Importantly, these challenges are not unique to the United Kingdom. Other Beveridge countries demonstrate similar patterns of policy ambiguity and regional variability. In Spain, CPs are not explicitly included in the national portfolio of covered prosthetic services, resulting in inconsistent access pathways that are largely dependent on regional interpretation and individual clinical circumstances (18). Similarly, Italy relies on regionally administered programs without a unified national reimbursement framework, leading to variability in eligibility and financial support (19). In contrast, Norway offers more clearly defined national coverage through its welfare system, though reimbursement limits may not fully offset the cost of higher-quality prostheses (20). Together, these examples illustrate that while Beveridge systems may formally recognize CPs within public healthcare structures, the degree of standardization and accessibility varies considerably across countries.

Across Beveridge systems, a central theme emerges: formal inclusion of CPs in healthcare policy does not guarantee equitable access. Instead, access is shaped by regional policies, administrative complexity, and diagnosis-based eligibility criteria. As a result, many patients encounter barriers even within systems designed to provide universal care. Nevertheless, to address these gaps, nonprofit and community organizations play a critical role in supplementing access. For instance, charity wig banks and organizations such as Macmillan Cancer Support and Cancer Hair Care provide financial assistance, education, and navigational support for individuals with hair loss, particularly for patients who do not meet strict eligibility criteria or who face administrative barriers within public systems (23, 27, 28).

In summary, Beveridge healthcare systems demonstrate that centralized policy frameworks can support recognition of CPs as medically relevant interventions; however, regional variation, administrative burden, and diagnosis-specific limitations are real-world factors that often undermine equitable access. These findings highlight a broader disconnect between policy inclusion and real-world accessibility, a theme that persists across healthcare models.

### 3.2 Bismarck

The Bismarck healthcare model provides insurance through a government-regulated compulsory insurance model in which a single federal pool of funds distributes risk-adjusted payments to multiple non-profit insurers (16). These insurers, often referred to as “sickness funds,” then disperse money to pay providers and cover costs for insured patients. The federal pool is primarily funded by contributions from employers and employees through payroll deductions, with minor tax contributions. Notably, the distribution, expenditure, and coverage of services offered by these funds are tightly regulated by the federal government, with slight variation in contribution rate and service quality between insurers. Countries such as France, Germany, Japan, and the Netherlands incorporate elements of this model, though most function as hybrid systems that blend Bismarck and Beveridge features (Table 2) (29, 30).

**Table 2:** Cranial prosthesis coverage in countries with Bismarck healthcare systems.

Country	CP policy	Eligibility	Reimbursement	Variation	Key limitations
France^a^	Included under SHI	Diagnosis-based; Long-term illness diagnoses covered more comprehensively	Dependent on Class of CP Yearly renewal	Coverage varies with illness categorization; Variation in supplemental insurance coverage	Reimbursement is capped; Medical prescription required; Premium CPs often require OOP payment
Germany^b^	Included under SHI; CP may be covered as medical aid on case-by-case basis	Typically requires physician prescription; coverage often more reliable for disease-related hair loss	Amount varies by insurer, device type, and approval Renewal period length not specified	Variation across SHI funds and between SHI vs. private insurance exists	Coverage may be inconsistent; prior authorization may be required; human hair CPs have higher out-of-pocket costs
Netherlands^c, d^	Citizens under Dutch basic health insurance entitled to CP reimbursement	People who have lost hair due to illness or medical treatment; prescription required	Max statutory reimbursement−482.50 Euro–subject to annual deductible Yearly renewal	Variation in supplemental insurance coverage	Reimbursement is capped; Medical prescription required; androgenetic alopecia not covered

^a^French Statutory Health Insurance CP reimbursement policy (32); ^b^German Statutory Health Insurance medical air and wig policies (36); ^c, d^Dutch health insurance reimbursement policies for CPs (29, 30).

Within Bismarck systems, CP coverage spans a broad spectrum ranging from relatively standardized and diagnosis-based reimbursement, to highly variable and insurer-dependent models. France represents one of the most structured approaches with its Statutory Health Insurance (SHI) model, which provides comprehensive universal care while supplemental private insurance fills remaining gaps (31). Under the French SHI system, CP coverage is broadly available for all French citizens experiencing hair loss due to “illness or its treatment,” regardless of patient age or extent of hair loss (32). Importantly, though all individuals with provider-diagnosed medical hair loss qualify for some degree of coverage, it is worth noting that such coverage is diagnosis-dependent. For instance, patients with conditions categorized as “exempt long-term illnesses” – such as malignant tumors or alopecia universalis—may be completely covered for CPs. However, individuals with diseases not considered “long-term illness,” such as androgenetic alopecia, may be subject to standard, yearly-renewed coverage caps (32). Once a diagnosis is determined, reimbursement of CPs in France is standardized and tiered based on prosthesis type, with full or partial coverage available for synthetic and mixed-fiber CPs, while higher-cost human hair prostheses are typically excluded or only partially reimbursed. This nationally defined structure promotes predictability in eligibility and reimbursement, which may help alleviate the CP-associated financial and emotional burden previously documented among French individuals with alopecia, although patients may still incur out-of-pocket expenses depending on prosthesis type and supplemental insurance coverage (33).

In contrast to France, Germany demonstrates a more decentralized implementation of CP coverage within a more traditionally Bismarck framework. In Germany, SHI covers approximately 90% of the population, while private health insurance (PHI) covers higher-income individuals, civil servants, and self-employed individuals (34). Much like SHI in France, the German system is financed primarily through employer-employee payroll contributions with risk-adjusted redistribution across sickness funds. Under German policy, CPs are eligible for reimbursement if deemed medically necessary by a prescribing physician; however, there is no standardized uniform reimbursement schema like that which France has (35). Rather, reimbursement is determined on a case-by-case basis and is often insurer-dependent. Coverage amounts, documentation requirements, and copayment guidelines vary widely across sickness funds (36). Synthetic CPs are more commonly covered within defined limits, while human hair CPs often require substantial patient contribution (37). This insurer-level variability has tangible financial implications (38). In fact, in a recent study examining the economic impact of AA in Germany found that CPs were the highest monthly out-of-pocket expense for individuals with AA, exceeding that of physician visits, pharmacotherapy, and other treatments (39).

The contrast between France and Germany highlights a key distinction within Bismarck systems: the degree of centralization in reimbursement policy significantly influences the patient experience. More standardized systems, such as France, offer clearer eligibility criteria and reimbursement expectations, whereas decentralized models, such as Germany, introduce variability that can increase financial burden for patients. Another pattern that is notable across Bismarck systems is that CPs are generally recognized as medically relevant, but are not uniformly prioritized within benefit structures. A part of this variability may be secondary to the structure of the Bismarck system promoting variability between sickness funds. However, as with other healthcare models, oncology-related hair loss is more consistently covered under Bismarck than primary dermatologic or autoimmune conditions. Additionally, patients frequently rely on supplemental insurance or external support programs to offset remaining costs. Thus, while the Bismarck system may offer more formalized pathways to CP reimbursement compared to other models, variability at the insurer level and incomplete financial coverage remain persistent barriers to equitable access.

### 3.3 National health insurance

National Health Insurance (NHI) is a universal, government-run insurance system funded primarily through taxes (40). This model provides universal coverage while maintaining largely private delivery of healthcare services, enabling centralized negotiation of healthcare costs while preserving provider autonomy. Countries such as Canada, Australia, and South Korea exemplify this healthcare model, though implementation varies at the regional and provincial level (Table 3) (41–43). Of these countries, Canada has both the most classic application of this model and the most available information regarding CP access (40).

**Table 3:** Cranial prosthesis coverage in countries with National Health Insurance systems.

Country	CP policy	Eligibility	Reimbursement	Variation	Key limitations
Canada^a^	Not explicitly included in national health benefits	Physician prescription may support case-by-case reimbursement; often oncology-related	No standardized national reimbursement; may be partially covered by private insurance or tax credits Wig lending programs offer 1 wig at a time for free indefinitely until they want to exchange	High; varies by province, insurance, and institutional programs	Lack formal policy; reliance on private insurance and charities
Australia^b^	Limited explicit CP inclusion in public systems	Typically restricted to specific programs or clinical contexts, such as oncology	Partial reimbursement through select government or private pathways Renewal period length not specified	Moderate to high; depends on state programs and private coverage	No unified national framework; inconsistent access
South Korea^c^	Limited public documentation of CP coverage	Reimbursement more commonly associated with medical necessity documentation	Partial or case-based reimbursement Renewal period length not specified	Moderate; varies by insurer and clinical context	Lack of standardized policy guidance

^a^Canadian Cancer Society wig and headwear support resources (41); ^b^Cancer Council Australia wig and headwear guidance (42); ^c^NPR report on South Korean CP policy developments (43).

In contrast to Beveridge and Bismarck systems, CP coverage within NHI frameworks is often notably absent from explicitly defined benefit packages and insurance pathways. Canada illustrates this limitation. While medically necessary physician and hospital services are universally covered under the Canada Health Act, prior attempts to expand the coverage to CPs has failed due to the categorization of CPs as fashion accessories (44). As a result, access to CPs is not guaranteed and instead depends on alternative pathways, including private insurance, employer-sponsored benefits, or case-by-case reimbursement following physician prescription or through the federal Medical Expense Tax Credit pathway. Such uncertainty results in significant costs for individuals with hair loss of any etiology, potentially contributing to decreased quality of life measured in Canadians with hair loss (45).

This absence of codified coverage shifts the burden of access to supplementary systems. Many patients obtain CPs through private insurance plans, some of which include coverage for medical CPs. However, most private insurance plans in Canada set lifetime caps of as little as $500 (46, 47). Further, aside from private insurance, others rely on tax-based reimbursement mechanisms or charitable programs like Ontario Works program or the Pink Heart Fund to fill coverage gaps left by Canadian legislation and the private market (48). Publicly funded cancer agencies and nonprofit organizations play a particularly prominent role in facilitating access, especially for individuals undergoing chemotherapy (49). For patients with non-oncologic hair loss, such as AA, access is far more variable and often depends on community-based resources, such as wig banks and advocacy groups (50).

Canada's CP policy landscape reflects a broader pattern within NHI systems: a lack of formal policy recognition translates to heavier reliance on diverse yet fragmented access pathways. Unlike Beveridge systems where CPs may be formally included but inconsistently implemented, or Bismarck systems where reimbursement is often defined but incomplete, NHI systems frequently omit CPs from statutory benefits altogether. This omission highlights the fundamental issue of the lack of consensus in deeming CPs as medically necessary devices within these frameworks.

### 3.4 United States

The United States operates under a structurally distinct healthcare system and, therefore, warrants separate analysis and discussion. Unlike the Beveridge, Bismarck, and NHI frameworks, the United States uses a mixed public-private system without universal coverage. Under this model, private insurance covers approximately two-thirds of the population, and public programs of Medicare and Medicaid provide coverage to specifically eligible groups (51). In this fragmented landscape, there is no federal mandate requiring coverage of CPs for medical hair loss. Thus, coverage is highly dependent on payer type, plan, and state-level legislation.

Medicare does not routinely classify CPs as durable medical equipment (DME) and generally excludes them from coverage. Medicaid coverage varies state by state, reflecting state-level discretion in defining optional benefits (52, 53). Within the private market, reimbursement may be available when wigs are prescribed as CPs and supported by documentation of medical necessity. Claims are commonly submitted using the HCPCS billing code A9282, primarily for private-insurer billing. However, use of this code does not guarantee coverage, given that Medicare routinely treats it as non-covered. Further, reimbursement policies, approval requirements, and benefit limits vary substantially across insurers (Table 4) (48). As a result, many patients incur significant out-of-pocket costs, which can vary depending on region and insurance type. In a recent US study of individuals with AA, CPs constituted the greatest category of out-of-pocket expenditure, with a mean reported cost of $930 +/- $1940 (54).

**Table 4:** Overview of cranial prosthesis coverage in the United States at federal, state, and private levels.

Country	CP policy	Eligibility	Reimbursement	Variation	Key limitations
United States (Federal)	No federal mandate for CP coverage	Requires physician prescription; often framed as “cranial prosthesis” for reimbursement	Medicare generally excludes CPs	Very high; dependent on payer type	Lack of federal standardization; classification ambiguity
United States (State level)	Some states mandate CP coverage	Typically includes hair loss due to chemotherapy, radiation, or alopecia	Coverage varies by state; may include reimbursement caps which renew annually	High; state-by-state variability	Inconsistent legislation; regional variation may drive inequities
Private Insurance	May cover CPs as prosthetic devices	Requires documentation of medical necessity and billing codes	Varies by plan, but generally offers reimbursement with caps Renewal period lengths vary by provider	Very high; insurer-dependent	Administrative burden; inconsistent approval

In the absence of federal standardization, CP coverage in the United States has largely evolved through state-level legislation and insurance regulation (Table 5) (55, 56). The Affordable Care Act (ACA) established Essential Health Benefits (EHBs) that must be covered by certain insurance plans; however, CPs are not specifically designated as a required EHC category, limiting reimbursement (57, 58). As a result, states seeking to expand CP coverage have largely relied on legislative mandates requiring insurers to provide coverage for eligible patients (59, 60). Namely, Massachusetts requires coverage of CPs for hair loss resulting from cancer or leukemia treatment when deemed medically necessary (61, 62). More recently, Illinois enacted legislation requiring many insurance plans to cover at least one CP every 12 months for hair loss due to alopecia, chemotherapy, or radiation treatment (63, 64). Although Illinois' expanded coverage requirements have taken effect, implementation and patient outcome data are not yet available. Nonetheless, these policies illustrate how state-level action has emerged as the primary mechanism for expanding CP access in the absence of a comprehensive federal reimbursement framework.

**Table 5:** State-level cranial prosthesis policy activity in the United States.

State	CP policy status	Eligibility	Reimbursement	Key developments	Key limitations
Massachusetts^a, b^	Established mandate	Coverage for cancer or leukemia patients only	Capped reimbursement of $350 annually	One of the earliest states to adopt CP coverage mandates	Limited to specific diagnoses; capped coverage
Minnesota^b, c^	Established mandate	Cancer and alopecia both covered	Capped reimbursement of $1,000 annually; one CP covered per year with physician's prescription	Notable for including autoimmune hair loss in coverage before cancer-related hair loss	Tedious administrative process for patients, with many required documentation elements
Illinois^d^	Recently expanded legislation as of 1/1/2026	Coverage for chemotherapy, alopecia, and radiation	Capped reimbursement of $1,000 annually	Recent legislation has expanded access and eligibility; major providers have updated policies to reflect this	Does not cover standard male or female pattern baldness; does not cover Medicaid or self-insured plans
California^e, f^	Legislation proposed (AB 2,668) but held under submission since May 2024	Would expand eligibility to diagnosed health conditions, chronic illness, or medical hair loss broadly	Maximum yearly coverage varies by payer	Failed to pass; minimal progress in recent years to expand CP coverage	Not yet enacted; cost concerns stalled progress
New York^e, g^	New legislation proposed (S4961) but not yet passed	Cancer and alopecia both covered; requires physician's prescription	Capped reimbursement of $750 annually	Newly introduced legislation is currently in committee senate; would expand insurance coverage to any health condition, chronic illness, or injury	Not yet enacted

^a^Massachusetts General Law governing insurance coverage of CPs (61); ^b^Ezennia et al., study on insurance coverage variability and out-of-pocket costs for CPs in AA (62); ^c^Minnesota statute for CPs for medical hair loss (56); ^d^Illinois state legislation expanding CP insurance coverage (64); ^e^National Alopecia Areata Foundation report on proposed CP policy in CA and NY (67); ^f^California Assembly Bill A2668 (65); ^g^New York Senate Bill S4961 (55).

Despite these hopeful efforts, some legislative efforts have also stalled. In California, for instance, Assembly Bill 2,668, introduced in 2024 with support from dermatologic and oncologic advocacy groups, proposed mandated insurance coverage for CPs (65). However, following a projected $29.5 million discal impact analysis by the California Health Benefits Review Program, the bill was held under submission in committee. At the federal level, the Wigs as Durable Medical Equipment Act has been introduced to classify CPs as covered prosthetics under Medicare. However, it has not been enacted (66).

Much like other countries operating under other frameworks, the gaps in coverage that have resulted from this highly variable coverage landscape have been partially mitigated by nonprofit and advocacy organizations. For instance, the National Alopecia Areata Foundation provides insurance navigation guidance and policy advocacy resources (67). Further, the American Cancer Society and charity groups such as Locks of Love facilitate CP donation programs and financial assistance, particularly for oncology patients and pediatric populations (68, 69). Nevertheless, these programs function outside of formal reimbursement structures and do not fully stand in for standardized insurance coverage.

Interestingly, the United States insurance landscape for CPs reflects a convergence of patterns observed across the other previously described healthcare systems. For instance, elements of regional variability seen in Beveridge systems are mirrored in state-level differences in coverage, while insurer-dependent reimbursement in private systems parallels the variability in Bismarck systems. Additionally, the absence of a universal mandate for CP coverage resembles the critical gap in the NHI framework, where CPs are not consistently included in statutory benefits. As a result, the United States healthcare system embodies a combination of these structural features, combining partial codification with significant fragmentation and administrative complexity. This convergence amplifies patient burden, as individuals must navigate multiple overlapping sources of variability, including insurance type, geographic location, and local policy.

### 3.5 Out-of-pocket

In the out-of-pocket (OOP) healthcare system, medical services are paid for directly by consumers, with little to no contribution from insurance or government programs (16). Notably, no country operates exclusively under an OOP model. However, several countries—primarily lower and middle-income countries—rely on OOP payments for the majority of total healthcare expenditures (70, 71). For instance, countries such as Nigeria and Yemen have reported OOP spending that exceeds 70% of national healthcare expenditures, whereas countries such as the UK and US report OOP expenditures of 9.7% and 11.4%, respectively (70).

In countries with a predominant OOP healthcare system, access to medical services is largely determined by individual ability to pay (Table 6). Consequently, resources put toward provision of healthcare typically prioritize essential services, such as infectious disease prevention, maternal-child health, and basic cancer care (71–75). This resource provision in turn leaves limited infrastructure or public financing means for supportive or quality-of-life measures. Given that hair loss is generally a benign process, CP coverage is often not included in these efforts. In fact, upon literature review, no published national policies or statutory guidelines were identified that address coverage of CPs for medical hair loss in primarily OOP systems.

**Table 6:** Cranial prosthesis coverage in countries with out-of-pocket healthcare systems.

Country	CP policy	Reimbursement	Variation	Key limitations
Nigeria	No published national CP policy or guidelines identified	CPs obtained primarily through private purchase, without reimbursement pathways	Very high; dependent on individual financial capacity and insurance access	Absence of policy and support systems; large socioeconomic disparities
Yemen	No published national CP policy or guidelines identified	CPs obtained primarily through private purchase, without reimbursement pathways	Very high; dependent on individual financial capacity and insurance access	Absence of policy and support systems; large socioeconomic disparities
India	No published national CP policy or guidelines identified	CPs obtained primarily through private purchase, without reimbursement pathways	Very high; dependent on individual financial capacity and insurance access	Absence of policy and support systems; large socioeconomic disparities
Philippines	No published national CP policy or guidelines identified	CPs obtained primarily through private purchase, without reimbursement pathways	Very high; dependent on individual financial capacity and insurance access	Absence of policy and support systems; large socioeconomic disparities

In such countries, CPs and cosmetic accessories for hair loss are obtained primarily through private purchase, without reimbursement pathways. Unlike in Beveridge and Bismarck systems, which offer CP access through relatively well-defined pathways, CP accessibility in OOP-dominant systems is largely a matter of affordability rather than policy eligibility. Compared to other healthcare models, OOP systems represent the most extreme manifestation of a pattern observed globally: when CPs are not formally recognized as medically necessary, access becomes inequitable and financially prohibitive. While other systems may partially mitigate this burden through insurance coverage or charitable support, such mechanisms are often limited or absent in OOP-dominant settings.

## 4 Discussion

CPs serve as an undeniable source of support and normalcy for individuals experiencing medical hair loss. However, across healthcare systems, the principal challenge is not whether CPs are recognized in policy, but whether that recognition translates into meaningful access. Although several countries have established reimbursement pathways, coverage is often partial, diagnosis-dependent, regionally variable, or administratively burdensome. Consequently, patients often continue to rely on charitable organizations, institutional programs, and community resources to offset costs and navigate the reimbursement process. These findings suggest that the effectiveness of CP policy is determined not only by the presence of coverage, but also by the clarity, consistency, and implementation of that coverage in practice.

Closely linked to this difficulty is the role of fragmentation in shaping the patient experience. Across healthcare systems, variability in CP coverage appears to be a key driver of inequitable access. However, the sources of this variability differ by model. In centralized systems, such as Beveridge frameworks, fragmentation often arises at the regional or institutional level. However, in decentralized systems such as Germany and the United States, variability is often driven by insurer or state-level decision making. This fragmentation introduces uncertainty, administrative burden, and increased financial responsibility for patients. Interestingly, the United States reflects a combination of these patterns, with a mix of regional variability, insurer-dependent reimbursement, and incomplete policy codification within one single system. In general, greater policy fragmentation is associated with increased patient expenses and administrative complexity, highlighting the importance of coordinated and transparent coverage frameworks.

The tangible consequences of this fragmentation are increasingly evident. For instance, in the United States, studies have demonstrated that CPs represent the largest category of OOP spending among individuals with AA, with a mean reported cost of roughly $1,000 (54). These findings are bolstered by further studies that have found that patients with moderate to severe AA have significant disruption of their daily lives due to time spent on concealment, altering major life decisions due to hair loss, and accruing OOP costs due to psychotherapy and CPs (76, 77). These findings are not singular to the United States, and similar studies in the Germany and England have found that patients with hair loss experience disproportionate rates of alienation, isolation, and strain due to high monthly costs (39, 78). Collectively, these findings suggest that fragmentation affects not only administrative complexity, but also the financial burden and lived experiences of patients seeking care.

Beyond structural variation, cultural values and diagnosis-specific considerations may play a significant role in shaping CP access. Although stigma associated with hair loss appears to be driven more by its visibility than its etiology, social and policy responses may differ depending on the perceived severity or legitimacy of the underlying condition (79). Across all systems, oncology-related hair loss seemingly receives more consistent coverage than autoimmune or scarring alopecia, reflecting broader societal emphasis on cancer survivorship and integration of supportive care within oncology pathways. For example, in the United States, CP coverage is frequently discussed within oncology survivorship guidelines, and state-level reimbursement mandates have historically focused on cancer-related hair loss (12, 80). Similarly, municipal subsidy initiatives in Japan specifically support chemotherapy-induced alopecia but not non-oncologic alopecia (2). Indeed, non-oncologic conditions such as AA are often excluded from comparable benefit structures despite equivalent psychosocial burden. This disparity extends beyond reimbursement policies and is reflected in broader healthcare systems. In Germany, for instance, AA has been described as a “lifestyle disease” within statutory insurance frameworks, limiting reimbursement for approved therapies and placing greater financial burden on patients (38). Thus, it is evident that non-oncologic hair loss receives inferior coverage and formal recognition compared to oncologic hair loss.

These trends raise important ethical questions about how medical necessity is defined and operationalized. While cancer-related alopecia is unquestionably worthy of supportive care, growing evidence demonstrates that the psychosocial consequences of non-oncologic hair loss are similarly profound. However, eligibility for reimbursement is frequently determined by diagnosis rather than patient burden, creating situations in which individuals with comparable impairments in quality of life receive markedly different levels of support. This discrepancy suggests that current coverage frameworks may be reflecting societal perceptions of disease severity rather than objective patient needs. Thus, future policy efforts should incorporate standardized assessments of psychosocial burden and medical necessity to ensure coverage better reflects and respects the lived experiences of individuals with hair loss.

Cultural perceptions of disease severity and medical necessity may further influence how CP policies are implemented and supplemented at institutional and community levels. Although hair loss is associated with psychosocial distress across populations, the cultural significance of hair varies considerably by region, gender, ethnicity, and religious tradition. Anthropological studies have demonstrated that hair often serves as a meaningful marker of femininity, cultural heritage, social belonging, and personal identity. Thus, the impact of hair loss can extend far beyond the physical realm. For instance, studies among Black women in both the United States and South Africa have highlighted the central role of hair in cultural tradition and identity, while research from Japan has documented high levels of stigma, embarrassment, anxiety, and depression among individuals with AA (81–84). These differences may influence how patients, clinicians, and policymakers perceive the severity of hair loss and, ultimately, whether CPs are viewed as medically necessary supportive devices or primarily cosmetic interventions.

Importantly, even in countries with clearly defined reimbursement pathways, external organizations remain essential in bridging gaps left by formal policy. In both France and the United Kingdom, national frameworks provide structured reimbursement for CPs, yet patients frequently depend on charitable foundations and community-based organizations to offset remaining costs, expand prosthesis options, or navigate access barriers. Conversely, countries with limited formal CP legislation may still achieve relatively consistent CP access through strong institutional support systems. Canada demonstrates this dynamic, as provincially administered cancer agencies and nonprofit networks work together to provide CPs and financial assistance despite the absence of explicit national CP coverage. These findings demonstrate that CP access is shaped not only by formal policy, but also by the interaction between healthcare systems, cultural attitudes, and community-level infrastructure. While nonprofit and institutional support systems play a critical role, their widespread necessity highlights the insufficiency of existing coverage frameworks and the need for more comprehensive and standardized policy solutions.

Each healthcare model demonstrates distinct strengths and weaknesses in its approach to CP coverage (Table 7). However, rather than identifying a single superior model, this review demonstrates that each healthcare system faces a common challenge: balancing accessibility, affordability, and administrative feasibility. Differences in financing structure shape how CP coverage is delivered, but no system fully eliminates barriers to access. Accordingly, policy discussions may benefit less from attempts to replicate any one national model, and more from identifying transferrable strategies that improve consistency, transparency, and patient-centered access across diverse healthcare environments.

**Table 7:** Strengths and limitations of healthcare models in CP coverage.

Healthcare model	Strengths	Limitations
Beveridge	National recognition of CPs; publicly funded access pathways	Regional variability; incomplete coverage; diagnosis-based restrictions
Bismarck	Structured reimbursement pathways; physician-driven eligibility	Insurer-level variability; incomplete reimbursement caps
National Health Insurance	Universal baseline healthcare coverage; strong institutional networks (ex: cancer agencies)	CPs often excluded from statutory benefits; inconsistent access; reliance on private insurance and charities
United States (mixed)	Flexibility for policy innovation; potential for private coverage pathways	High degree of fragmentation; state and insurer variability; high out-of-pocket costs; administrative burden
Out-of-pocket	No complex administrative barriers	No formal coverage; affordability-driven access, leading to significant inequity; lack of policy recognition

Our findings also highlight several important research gaps. Specifically, there is a scarcity of standardized, international data on CP utilization, access pathways, and patient outcomes, limiting the ability to directly compare the effectiveness of different healthcare models. Though the economic burden of CP acquisition has been explored in some select studies, it remains incompletely characterized across diverse healthcare systems, specifically for patients with non-oncologic hair loss. Additionally, patient outcomes such as quality of life, satisfaction with access pathways, and psychosocial impact are underrepresented in policy research. These limitations are especially evident in countries where CP policies have received little to no formal investigation. For example, despite a substantial burden of alopecia and a large national healthcare system, published literature regarding CP accessibility and reimbursement in China remains sparse (85). Recent qualitative work has highlighted financial barriers to obtaining CPs and other hair camouflage methods, as well as limited clinical counseling regarding available resources (86, 87). However, little is known about formal reimbursement pathways or the role of public insurance in facilitating access. This lack of data in one of the most populous countries in the world with notable alopecia burden underscores a broader challenge in the field: for many countries, the absence of published evidence may make it difficult to distinguish between true policy gaps and gaps in policy visibility. Addressing these gaps through comparative health policy studies and qualitative research may be an essential next step to inform evidence-based improvements in CP coverage.

Looking forward, several potential developments may help advance this field. Expanding diagnostic eligibility criteria to include both oncologic and non-oncologic hair loss could improve equity in CP access. Further, establishing minimum reimbursement thresholds may reduce financial burden and increase consistency across healthcare systems. Equally important is simplifying administrative pathways for CP reimbursement, as this could further improve usability and reduce barriers to care. At a broader level, increasing standardization of CP coverage—whether at the state, national, or institutional levels—may enhance predictability and reduce disparities, though the optimal approach will likely vary by system and country. Strengthening partnerships between healthcare systems and nonprofit organizations may also provide a practical and scalable mechanism to improve access in the near future. Finally, development of standardized frameworks for assessing medical necessity may help align coverage decisions with patient burden rather than diagnosis alone, creating a more equitable and evidence-based foundation for future policy.

## 5 Conclusion

In this narrative review, we highlight substantial global variability in CP coverage for medical hair loss, shaped largely by underlying healthcare financing structures. We illustrate that, although several countries have established pathways for CP coverage, substantial gaps persist, particularly for individuals with non-oncologic hair loss and those navigating fragmented or inconsistently implemented policies.

Moving forward, greater recognition of CPs as medically necessary supportive devices may serve as a critical foundation for expanding equitable access. The development of standardized frameworks for assessing medical necessity, alongside clearer eligibility criteria and reimbursement guidelines, could help reduce variability in coverage and align policy with the documented psychosocial impact of hair loss. Continued research, policy development, and advocacy efforts are essential to ensure that access to CPs reflects the needs of patients worldwide.
